# Nanofiller Dispersion, Morphology, Mechanical Behavior, and Electrical Properties of Nanostructured Styrene-Butadiene-Based Triblock Copolymer/CNT Composites

**DOI:** 10.3390/polym11111831

**Published:** 2019-11-07

**Authors:** Ulrike Staudinger, Bhabani K. Satapathy, Dieter Jehnichen

**Affiliations:** 1 Leibniz-Institut für Polymerforschung Dresden e.V., Hohe Str. 6, 01069 Dresden, Germany; djeh@ipfdd.de; 2 Department of Materials Science and Engineering, Indian Institute of Technology Delhi, Hauz Khas, New-Delhi 110016, India; bhabaniks@googlemail.com

**Keywords:** block copolymer, carbon nanotubes, nanofiller dispersion, phase behavior, morphology, mechanical properties, electrical conductivity

## Abstract

A nanostructured linear triblock copolymer based on styrene and butadiene with lamellar morphology is filled with multiwalled carbon nanotubes (MWCNTs) of up to 1 wt% by melt compounding. This study deals with the dispersability of the MWCNTs within the nanostructured matrix and its consequent impact on block copolymer (BCP) morphology, deformation behavior, and the electrical conductivity of composites. By adjusting the processing parameters during melt mixing, the dispersion of the MWCNTs within the BCP matrix are optimized. In this study, the morphology and glass transition temperatures (*T*_g_) of the hard and soft phase are not significantly influenced by the incorporation of MWCNTs. However, processing-induced orientation effects of the BCP structure are reduced by the addition of MWCNT accompanied by a decrease in lamella size. The stress-strain behavior of the triblock copolymer/MWCNT composites indicate higher Young’s modulus and pronounced yield point while retaining high ductility (strain at break ~ 400%). At a MWCNT content of 1 wt%, the nanocomposites are electrically conductive, exhibiting a volume resistivity below 3 × 10^3^ Ω·cm. Accordingly, the study offers approaches for the development of mechanically flexible functional materials while maintaining a remarkable structural property profile.

## 1. Introduction

Block copolymers (BCPs) are self-organizing polymeric materials. Due to the thermodynamic incompatibility between covalently bonded blocks, the chain segments microphase separately into various nanostructures differing in their interfacial curvature. The phase behavior mainly depends on the copolymer architecture, the overall degree of polymerization, the block composition, and the interaction parameter between the chain segments. The combination of hard (A) and soft (B) components offers a broad spectrum of possibilities to design the material properties. Comparing AB diblock and ABA triblock copolymers with the same molecular weight, with the same composition, and that have developed the same morphology, the latter exhibits a superior mechanical property profile. For example, in styrene-butadiene-styrene (SBS) triblock copolymers the two outer polystyrene (PS) blocks are interconnected via the polybutadiene (PB) matrix (forming bridges of the soft molecular chains) and form a sort of physical cross-linking, which results in significantly higher strength, stiffness, and elongation of the triblock copolymer compared to their compositionally equivalent styrene-butadiene (SB) diblocks [1]. No effective physical network can form in the diblocks, since the elastomer molecules are only connected to the PS molecules at one end. Therefore, diblocks show relatively brittle behavior, which is mechanistically described by a cavitation model [2]. 

By replacing the butadiene middle block in SBS triblocks with a statistical SB copolymer and varying the PS outer block lengths, BCPs with very interesting property profiles can be designed ranging from thermoplastic elastomers with high elongation at break to very tough and stiff BCPs [3,4,5,6,7,8,9]. Another strong tool to adjust desired property profiles is to vary the molecular architecture of the BCPs such as in starblocks or multigraft copolymer architectures.

Due to their structural characteristics and interesting designable property profile, block copolymers offer high potential as template matrices for controlled and phase-selective incorporation of nano-scaled particles as the nanofiller dimensions are in the range of the domain sizes of the block copolymers. This opens up new possibilities to develop functional materials exhibiting specific magnetic [10,11,12], optical [13], electrical [14], or mechanical properties [15]. Selective localization of nanoparticles within one phase of the block copolymer nanostructure is strongly dependent on the geometrical and dimensional characteristics of the particles. Whereas spherical particles theoretically can localize within all possible morphologies, one-dimensional particles like carbon nanotubes (CNTs), having a high aspect ratio, can only localize in lamellar, cylindrical, or co-continuous structures when domain sizes of the block copolymer phases sufficiently exceed the size of the nanoparticles [16]. Via selective incorporation of CNTs, mechanical, electrical, or thermal properties of BCPs could be improved while significantly reducing not only the extent of filler incorporation but also the material costs compared to homogeneous filler dispersion within a polymer matrix. Using aligned BCP structures, nano-scaled electrical or thermal conductive paths could be generated selectively in one of the phases that could be a flexible matrix. Furthermore, the mechanical strength of BCPs could be increased without reducing their elasticity. The most significant challenge of using CNTs as nanofiller in BCPs is ensuring their sufficient dispersion and uniform distribution in the polymer matrix in spite of their high aspect ratio (length in micrometer scale) and intrinsic tendency to agglomerate.

Various approaches have been adopted to incorporate CNTs within BCPs to modify the BCP properties [17,18,19,20,21,22,23,24,25], but only a few studies have been able to successfully demonstrate selective CNT localization in one of the BCP phases [26,27,28,29,30]. A more detailed overview of these attempts is given in our previous publication [31]. It can be concluded that, preferably, controlled functionalization of the CNTs could facilitate their incorporation into BCPs with lamellar structures, leading to selective localization of the CNTs in the nano-scaled lamella [28,29,30]. More recently, Hasanabadi et al. [32] studied the structural behavior of a cylindrical SEBS triblock copolymer containing MWCNTs, which were modified with PS-brushes. The MWCNTs were found to be located exclusively in the PS domains, suggesting that the surface modification of the MWCNTs significantly improves the interaction and compatibilization with the BCP matrix.

The structure–property correlations of block copolymers on the basis of styrene and butadiene using different architectures like triblock or starblock; introducing a random styrene-butadiene middle block, asymmetric outer blocks, or even tapered block transitions; and varying the block composition have been intensively studied in the past [6,7,8,9,33,34,35,36,37,38,39,40,41]. On the basis of the well-known property characteristics of such BCPs, our previous study focused on the investigation of structure and the property development of a star-shaped styrene-butadiene-based block copolymer melt mixed with commercial non-functionalized multi-walled carbon nanotubes (MWCNTs) [31]. The co-continuous BCP morphology was not affected by incorporation of the MWCNTs, and no selective localization of the MWCNTs could be detected. However, the MWCNTs were homogeneously dispersed within the BCP matrix. The electrical percolation threshold was observed to be at ~1 wt%, and a significant increase in Young’s modulus was exhibited at a MWCNT content of 0.1 wt% while maintaining the elastomeric BCP property profile.

The focus of the present work is to study the CNT dispersion and localization behavior in a linear S-SB-S triblock copolymer with lamellar structure through the use of shortened MWCNTs, and its impact on morphology, mechanical behavior, and electrical properties. The BCP has been intensively investigated with respect to its phase behavior, morphology formation, and mechanical properties [5,36,37,42,43,44]. Its lamellar morphology makes it well suited for dispersing nanofillers such as one-dimensional CNTs.

## 2. Experimental

### 2.1. Materials

The block copolymer used in this study (named LN3) was provided by BASF AG Co (Ludwigshafen am Rhein, Germany). The synthesis is described by Knoll and Niessner [17]. LN3 is a linear triblock copolymer exhibiting highly asymmetric block architecture. The middle block is comprised of a random styrene-butadiene copolymer. The outer blocks consists of a short and a long PS arm. The molecular characterization of the individual components according to the information from the manufacturer is summarized in Table 1.

Multiwalled carbon nanotubes NC7000 from Nanocyl^TM^ (Sambreville, Belgium), shortened to about 35 % of the original length (~0.5 µm) by ball milling for 10 h [45], were used for this study. NC7000 were produced in an industrial, large-scale catalytic vapor deposition (CVD) process with an average diameter of ~9.5 nm, a carbon purity of 90 %, and a surface area of 250–300 m^2^/g [46]. Reportedly, NC7000 are characterized by good dispersability in various thermoplastic polymer melts [47,48], in aqueous dispersions [49,50], and in organic solvents [51] compared to other types of MWCNTs. The shortened type of NC7000 was used to facilitate the dispersion and localization of the MWCNTs within the nano-scaled domains of the block copolymer matrix.

### 2.2. Melt Mixing of S-SB-S/MWCNT Composites

Granules of LN3 were melt mixed with 1 wt% MWCNTs using a twin screw micro compounder DSM 15 (Xplore Instruments, Sittard, Netherlands) at varying temperatures of 180 °C and 200 °C and varying screw speeds of 100 rpm and 200 rpm each for 5 min. As reference, pure LN3 was also extruded using the lower screw speed of 100 rpm at 180 °C and 200 °C. The details of the processing conditions of each sample/composition are given in Table 2. The letters A, B, C, and D used in the samples’ names refer to the specific processing conditions. After characterizing the MWCNT macrodispersion of the composites, additional composites with 0.1 wt%, 0.3 wt%, and 0.5 wt% of MWCNTs and the pure LN3 were melt mixed using the processing parameters (D), which resulted in the lowest agglomerate area ratio, as described in Section 2.3.

The extruded strands were chopped and compression molded at 200 °C for 1 min on a hot press PW40EH (Otto-Paul-Weber GmbH, Remshalden, Germany) to obtain rectangular plates of 80 × 80 mm^2^ and a thickness of 0.5 mm.

### 2.3. Morphological Characterization

The state of macrodispersion of the MWCNTs in the composites was characterized by cutting about ten thin cross sections of 5 µm thickness from the extruded strands of each sample using a Leica RM 2265 microtome equipped with a liquid nitrogen freezing device LN22 (Leica Microsystems GmbH, Wetzlar, Germany) at 0 °C. The sections were transferred to glass slides and fixed with Aquatex^®^. Transmission light microscopy (TLM) was performed on the samples using an Olympus-BH2 microscope combined with a camera DP71 and connected to Stream Motion evaluation software (all from Olympus Deutschland GmbH, Hamburg, Germany). Ten representative images of each sample with dimensions of 600 × 800 mm^2^ at ten-time magnification were used to determine the CNT agglomerate area ratio (*A* in %). The detected area of non-dispersed CNTs (with equivalent circle diameters >5 µm, area >19.6 µm^2^) was divided over the entire sample area, and the mean and standard deviation values were calculated.

To study the block copolymer morphology, a transmission electron microscope LIBRA^®^ 120 (Carl Zeiss AG, Oberkochen, Germany) with an acceleration voltage of 120 kV was used. Ultrathin cross-sections of ~80 nm were cut at −80 °C from the extruded strands using an ultramicrotome EM UC6/EM FC6 (Leica Microsystems, Austria) with a diamond knife and transferred to carbon grids. The polybutadiene (PB)-rich phase was stained with osmium tetroxide to enhance contrast in the TEM image as the heavy metal absorbs electrons or scatters part of the electron beam. For sample LN3_1_D, an additional longitudinal cut lengthways to the extruded strand was prepared to study orientation effects of the composite morphology. The long period of the nanostructures and the domain sizes of the PS-rich and PB-rich phases were measured using Stream Motion evaluation software. Five TEM images of each sample were evaluated measuring about 40 lamellae per image to ensure a statistically well-founded validation of the measurement data. Additionally, samples without staining were investigated via TEM to visualize the CNT dispersion within the BCP matrix. These images do not show any contrast between the PS and PB phase in the BCP and the BCP morphology can not be visualized. However, the CNTs are clearly visible. In contrasted (stained) samples, the CNTs are more difficult to detect and only partially visible in the TEM images, depending on the focusing of the electron beam and the magnification as well as the CNT content in the sample. Therefore, the staining time was kept short (for 2 h) to ensure simultaneous visibility of CNTs and BCP structure.

**Figure 1:**
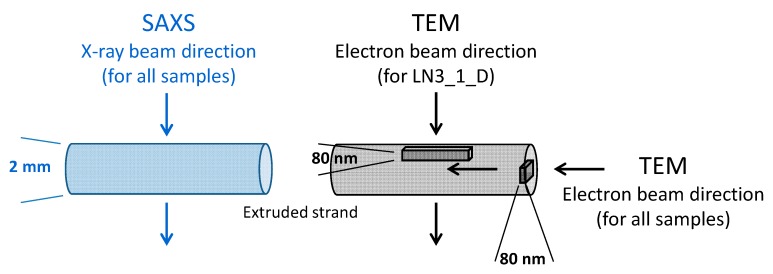
Sample preparation and beam direction in small-angle X-ray scattering (SAXS) and transmission electron microscopy (TEM) analysis.

Small-angle X-ray scattering (SAXS) was performed in transmission through the bulk material of the extruded strand (see Figure 1) using a multi-range device Ganesha 300 XL+ (SAXSLAB ApS, Kopenhagen, Denmark) equipped with a 2D-detector Pilatus 300K. 2D scattering patterns and radial intensity scattering curves were evaluated to analyze the type of structure, its orientation, and its long period.

### 2.4. Dynamic Mechanical Analysis.

The influence of MWCNT on the glass transition behavior of the block copolymer phases of the composites was characterized by conducting dynamic mechanical analysis (DMA) measurements using test specimens of compression-molded plates with dimensions of 0.5 × 30 × 8 mm^3^. Standard oscillatory temperature sweeps between −100 °C and 120 °C at a frequency of 0.1 rad/s and a strain amplitude of 0.01 % were performed using an ARES G2 Rheometer (TA Instruments, New Castle, DE, USA) in torsion mode. The storage modulus (G’), the loss modulus (G’’), and the damping coefficient (tan δ) were measured.

### 2.5. Evaluation of Electrical and Mechanical Properties

Electrical measurements were performed on the compression-molded plates of the composites using a Keithley electrometer E6517A (Keithley, Cleveland, OH, USA). For samples with volume resistivity > 10^7^ Ω·cm (open symbols in the graph), the electrometer was combined with a Keithley test fixture 8009 (Keithley, Cleveland, OH, USA). For conductive samples (volume resistivity <10^7^ Ω·cm), strips of 30 × 4 mm^2^ were cut from the plates and measured using a 4-point test fixture with gold electrodes having a distance of 16 mm between the source electrodes and 10 mm between the measuring electrodes, which were connected to the 6517A voltage source (filled symbols in the graph).

To investigate the stress-strain behavior of the composites tensile, tests were performed at room temperature using a Z010 Zwick universal testing machine (ZwickRoell GmbH & Co. KG, Ulm, Germany) equipped with a MultiXtens extensometer according to DIN EN ISO 527-2/S3a/50 at a crosshead speed of 50 mm/min. Dog-bone shaped specimens of 50 × 4 × 0.5 mm^3^ were cut from the compression molded plates. For each sample, five specimens were tested to confirm the statistical reproducibility of the mechanical properties.

## 3. Results and Discussion

### 3.1. Macrodispersion of MWCNTs

**Figure 2:**
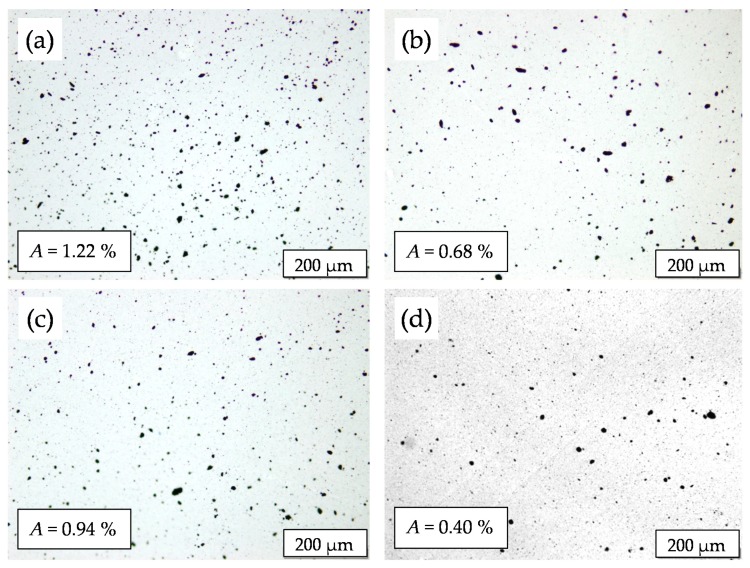
Transmission light microscopy (TLM) images and agglomerate area ratio (*A*) of melt mixed LN3/MWCNT composites with 1 wt% MWCNTs at varying processing conditions: (**a**) A: 180 °C, 100 rpm, (**b**) B: 180 °C, 200 rpm, (**c**) C: 200 °C, 100 rpm, and (**d**) D: 200 °C, 200 rpm.

To adjust suitable melt mixing parameters for optimized dispersion and distribution of the MWCNTs within the block copolymer melt, the processing temperature and screw speed were varied for composites containing 1 wt% of MWCNTs. The macrodispersion of the MWCNTs was evaluated on the ultrathin slices from the extruded strands for different mixing conditions using TLM as displayed in Figure 2. The letters A, B, C, and D in the sample designations refer to the specific processing conditions, as given in Table 2. The macrodispersion of the MWCNTs improves with temperature and screw speed, as indicated by the significantly decreased agglomerate area ratio (*A*) from *A* = 1.22 % (for processing condition A) to *A* = 0.40 % (for processing condition D). When comparing the evaluated *A*-values of processing condition B (*A* = 0.68 %) and C (*A* = 0.94 %), it can be assumed that the increase of screw speed from 100 rpm to 200 rpm has a more significant influence on the improved MWCNT dispersion than the increase of temperature from 180 °C to 200 °C. This assumption is also supported by the standard deviation values of *A* (displayed in Figure 10, which will be discussed in Section 3.3), which are distinctly lower for processing conditions B and D (higher scew speed) than for processing conditions A and C (lower screw speed). This implies the presences of larger remaining MWCNT agglomerates when applying lower screw speed, i.e., lower shear forces during melt mixing. Furthermore, on the basis of these results, composites with 0.1–0.5 wt% MWCNTs and the neat LN3 matrix (as reference) were processed under the condition D. The macrodispersion of MWCNTs in these composites was observed to be very good with values of *A* ≤ 0.07% (see Figure 10) and thereby indicating a good distribution and dispersion of the primary CNT agglomerates within the BCP matrix. It has to be considered that only agglomerates with diameter > 5 µm are included in the analysis of the agglomerate area ratio.

### 3.2. Block Copolymer Nanostructure and Phase Behaviour

The linear S-SB-S triblock copolymer used in this work exhibits a lamellar morphology. Its phase behavior and nanostructure have been investigated in former studies [5,37]. The outer PS-blocks act as glassy domains comprising ~50 wt% of the overall block copolymer composition, and the SB middle block acts as rubbery phase. This hard and soft phase composition ratio of 50:50 causes the formation of a lamellar morphology instead of a hexagonal morphology, which may theoretically be expected for linear SBS triblock copolymers having the same overall PS content of 75 wt% [1]. The long period of the lamellar structure and the domain sizes of the PS-rich and PB-rich phases have been found to be significantly dependent on the processing conditions. Average values of lamellar sizes are given in Table 3. It has to be noted that all samples show a broad distribution of the measured lamellar sizes, as shown by their standard deviations (Table 3). For example, long periods in the range of ~14 nm to ~46 nm for LN3_D were observed, as revealed from image analysis. Shear forces during the extrusion process result in considerable orientation effects of the molecular chains, indicated by stretched or widened lamellae. Such orientation effects are mainly responsible for the variations in long period and domain sizes of the PB-rich and PS-rich phases within the lamellar block copolymer morphology. Therefore, a detailed discussion about the size variations among the various samples should be treated with caution, and hence the influence of processing temperature and/or screw speed on the lamellae size cannot be evaluated.

**Figure 3:**
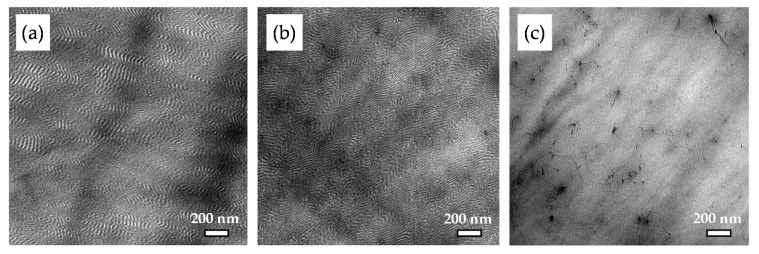
Morphology images from TEM of (**a**) LN3_A, (**b**) LN3_1_A, and (**c**) multiwalled carbon nanotubes (MWCNT) dispersion, in LN3_1_A (non-contrasted sample)**.**

Figure 3 displays the lamellar morphology of sample LN3_A. The addition of 1 wt% MWCNTs does not change the lamellar matrix morphology, as shown in the TEM image in Figure 3b. Figure 3c represents the local dispersion of MWCNTs within the LN3_A matrix on a non-contrasted sample. Such an observation may lead to the understanding that the incorporation of MWCNT (1 wt%) in the given processing conditions (A) does not alter the thermodynamic stability of the block copolymer nanomorphology.

**Figure 4:**
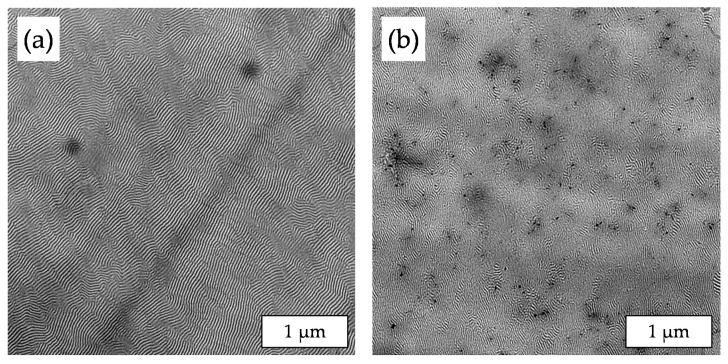
Morphology images from TEM of (**a**) LN3_C and (**b**) LN3_1_C.

However, the average lamellar long period thicknesses of the composites were measured to be significantly reduced, in contrast to their block copolymer matrix LN3 processed under identical conditions (processing conditions C and D). Such an observation is particularly striking in case of samples LN3_C and LN3_1_C, as revealed by their corresponding long periods (Table 3) as shown in the morphology images given in Figure 4.

**Figure 5:**
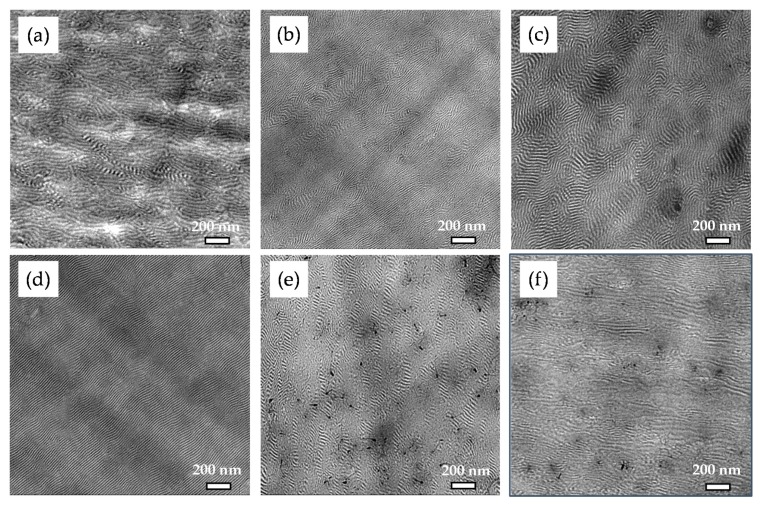
Morphology images from TEM of (**a**) LN3_D, (**b**) LN3_0.1_D, (**c**) LN3_0.3_D, (**d**) LN3_0.5_D, (**e**) LN3_1_D, and (**f**) LN3_D_1, (longitudinal view).

Figure 5 shows the morphologies of LN3_D and its corresponding nanocomposites. All samples show lamellar structures, whereby the orientation of the lamellae and the long-range order of the nanostructures are differently pronounced. For nanocomposites with MWCNT contents of 0.5 wt% and less, the MWCNTs are hardly visible in the images. In the TEM image of sample LN3_1_D, well-distributed MWCNTs penetrating the nanomorphology can be identified (Figure 5e). It is difficult to judge if or to what extent the CNTs are preferentially located in one of the BCP phases or at the interface. The TEM image in Figure 5f presents a longitudinal view into the extruded strand of LN3_1_D (see also Figure 1), showing significant orientation of the lamellae along the melt flow direction. The MWCNTs visible in the image do not show a preferred orientation direction. MWCNTs agglomerate with diameters smaller than 1 µm are distributed in the matrix and interfere with the morphology formation.

**Figure 6:**
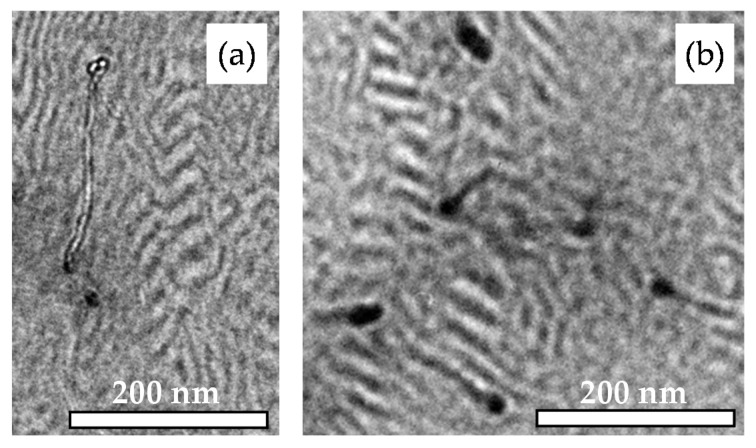
Magnified TEM images of sample LN3_1_D: (**a**) size comparison between the BCP domains and the MWCNTs and (**b**) visualization of the MWCNT localization in the BCP lamellas.

Figure 6 shows two enlarged TEM images of the morphology of sample LN3_1_D. Due to the size ratio between the BCP nanodomains and the MWCNTs, which is illustrated by Figure 6a, it is hardly possible to localize CNTs in one of the BCP phases, especially the longer MWCNTs. Figure 6b shows a magnified area of the BCP nanostructure in which single MWCNTs seem to be preferentially anchored in the PB-rich phase. However, the MWCNTs are located mainly across the BCP domains, as shown in Figure 5e, demonstrating that the MWCNTs, despite being shortened in a ball mill, are mostly too long to be localized in the PS or PB lamellas.

**Figure 7:**
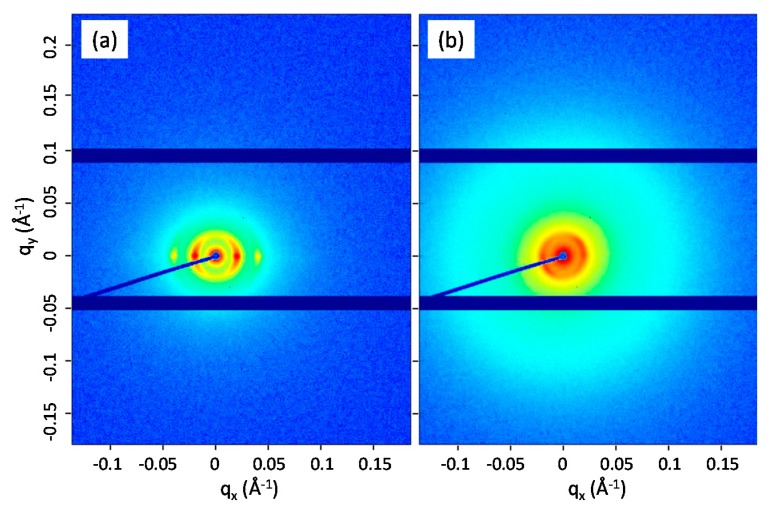
Small-angle x-ray scattering (SAXS) 2D scattering patterns of (**a**) LN3_A and (**b**) LN3_1_A.

**Figure 8:**
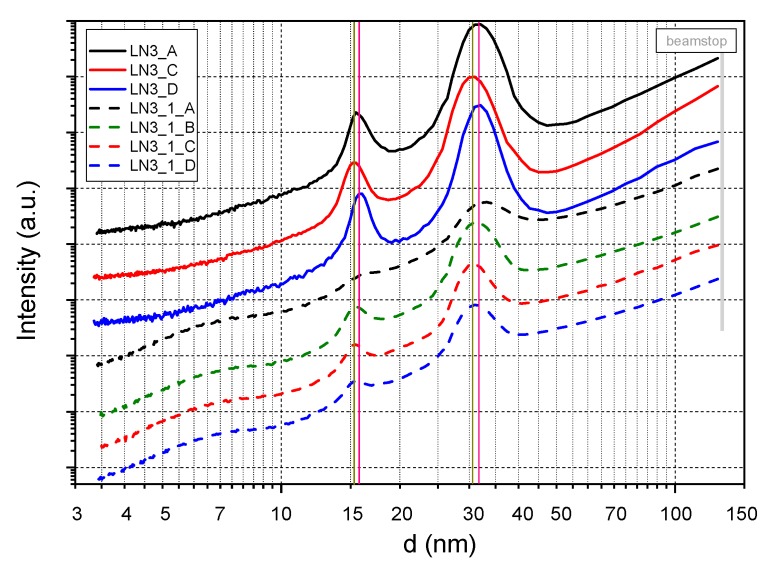
Radial scattering curves of pure LN3 and LN3 composites with 1 wt% MWCNT at processing conditions A (180 °C, 100 rpm), B (180 °C, 200 rpm), C (200 °C, 100 rpm) and D (200 °C, 200 rpm). The scattering curves were calculated from the related 2D-SAXS patterns by azimuthal integration within a small sector *Δϕ* = ±5° relative to the maxima direction of azimuthal intensity distribution.

The SAXS analysis was performed for the pure LN3 samples and the nanocomposites with 1 wt% MWCNT. Primary information of the scattering behavior is illustrated in Figure 7 as SAXS patterns. From the radial scattering curves in Figure 8, the formation of a lamellar morphology was confirmed for all samples exhibiting long periods between ~30 nm and ~32 nm. In contrast to the TEM images, no significant reduction of the long period could be observed with the addition of MWCNTs. The reason could be the different measuring ranges used for the calculation of the long period. While the TEM image analysis is performed in a range of a few µm^2^ area with a sample thickness of only 80 nm, X-rays with a beam diameter of about 0.5 mm irradiate through a sample thickness of about 2 mm (see Figure 1). This means that the SAXS analysis provides mean values of the long periods over the entire strand thickness, while the TEM images show the morphology of very local, high-resolution areas. For the pure LN3 samples, a significant orientation of the lamellar structure in the direction of the axis of the extrusion strand is detectable, as shown for LN3_A in Figure 7a. The degree of orientation reduces by the addition of the CNTs (Figure 7b). As shown by the radial intensity curves in Figure 8, the phase separation is enhanced by the addition of MWCNTs. Due to the presence of amorphous MWCNTs, an additional scattering maximum in the d-range of 4 nm to 10 nm occurs that is similar to non-phase separated behavior. Due to the particle scattering of the MWCNTs, the total scattering intensity of the composites is increased compared to the pure LN3 samples.

**Figure 9:**
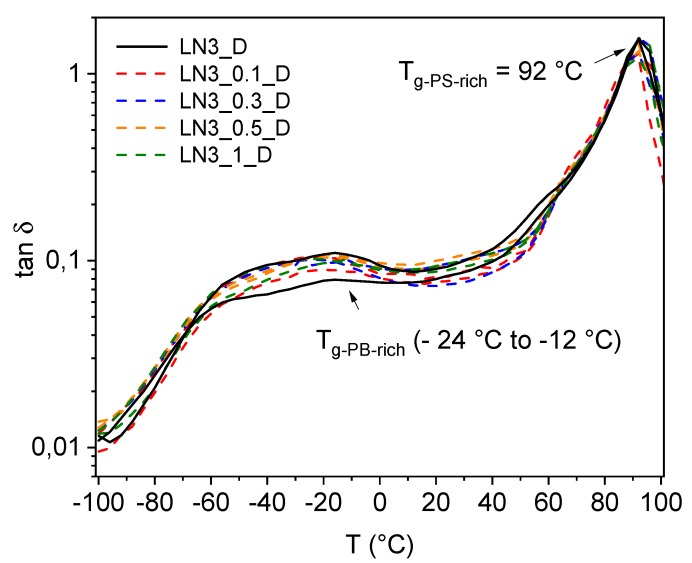
*tanδ* curves of LN3_D and its corresponding composites evaluated from DMA measurement.

From DMA measurements, the glass transition temperatures of the PB-rich and PS-rich phases of the LN3 block copolymers were evaluated. For the analysis, specimens were taken in horizontal and vertical directions from the pressed plates from each composition in order to take into account possible orientation effects of the lamellar structure as observed by TEM and SAXS. This means that for each composition, the curves of two specimens prepared perpendicular to each other are shown. To get a better overview, only the *tan* *δ* curves for neat LN3_D and composites melt processed under the same conditions (D) are plotted in Figure 9. The glass transition temperature of the PB-rich phase (mixed phase of styrene and butadiene) of all investigated samples remained lower than the theoretical values estimated using Fox equation [52] for a corresponding random SB copolymer (*T_g-SB_* = −37 °C). Such an observation indicated a partial miscibility of the SB middle block with the PS outer blocks. A broad peak-maximum rather than a sharp peak was observed for *T_g_* of the PB-rich phase, with maximum values ranging from -24 °C to −12 °C. Obviously, these variations are caused by the alignment of the polymer chains during the extrusion process. Comparing the two curves of one composition, which refer in each case to the specimens prepared perpendicular to each other, the *tan* *δ* curves show a clear dependence of the peak intensity of the PB-rich phase on the structural orientation. This is particularly pronounced in the case of sample LN3_D (black curves in Figure 9), where the peak intensities of both curves differ significantly from each other. The reasons behind such effects are not fully understood. However, the possibility of CNTs being aligned during the shear flow of the melt, which in turn may lead to higher peak intensity, should be considered.

No significant influence of CNT addition and processing parameters on *T_g_* could be determined. The *T_g_* of the PS-rich phase for the neat LN3 samples and composites remains unaffected in the form of a sharp peak at ~92 °C irrespective of processing types. Such an observation remained well in accordance with the phase behavior of previously investigated starblock copolymer/MWCNT-based composites [31].

### 3.3. Structure–Property Correlations (Electrical and Mechanical Properties)

**Figure 10:**
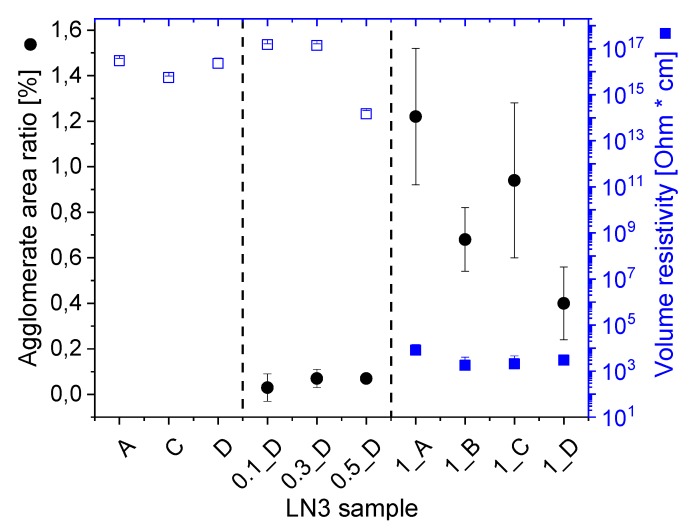
Agglomerate area ratio and volume resistivity of LN3 and LN3/MWCNT composites for processing conditions A (180 °C, 100 rpm), B (180 °C, 200 rpm), C (200 °C, 100 rpm) and D (200 °C, 200 rpm).

The electrical properties of the BCP/MWCNT composites are strongly dependent on the state of dispersion and electrical percolation of the nanofiller in the BCP matrix. As shown in Figure 10, composites with a filler content up to 0.5 wt% MWCNTs with very low agglomerate area ratios are non-conductive and thus indicate a well dispersed morphology with isolated MWCNTs distributed within the BCP matrix. Composites containing 1 wt% of MWCNTs are electrically conductive, having a very low volume resistivity of ~8.1 × 10^3^ Ω·cm when processed under condition A and ~1.8 × 10^3^ Ω·cm when processed under condition B. Sample LN3_1D exhibits the lowest agglomerate area of the conductive samples due to the optimized melt mixing parameters, such as a temperature of 200 °C and a screw speed of 200 rpm. The electrical volume resistivity of LN3_D was measured to be ~3.0 × 10^3^ Ω·cm. Thus, the percolation threshold for LN3/MWCNT composites remains between 0.5 wt% and 1 wt% of MWCNT content. Such a percolation threshold range is lower than in recently investigated melt-mixed MWCNT filled composites based on a styrene-butadiene starblock copolymer (3G55, Styrolution Group) such as the matrix with an overall PS content of 75 wt% and a complex wormlike morphology due to the presence of short, long, and highly curved lamellae [31]. In that system, another grade of MWCNTs named NC3150 from Nanocyl was used as a filler, which has higher carbon purity than NC7000 (~95 %) and with an average length of <1 µm. It is apparent from these observations that to reach low resistivity values like that in LN3/MWCNT composites, ~2 wt% of NC3150 may be necessary to add into the star block copolymer matrix.

**Figure 11:**
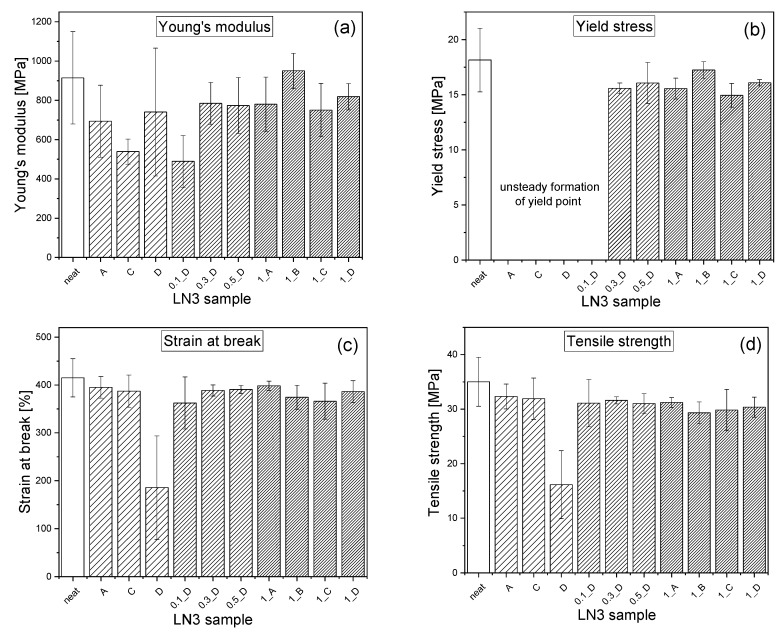
Mechanical characteristics of LN3 and LN3/MWCNT composites evaluated from tensile tests: (**a**) Young’s modulus, (**b**) Yield stress, (**c**) strain at break, and (**d**) tensile strength.

**Figure A1:**
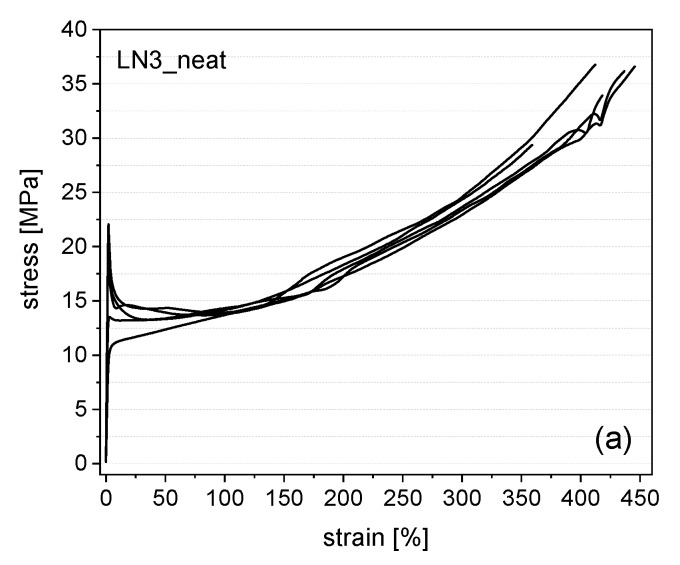

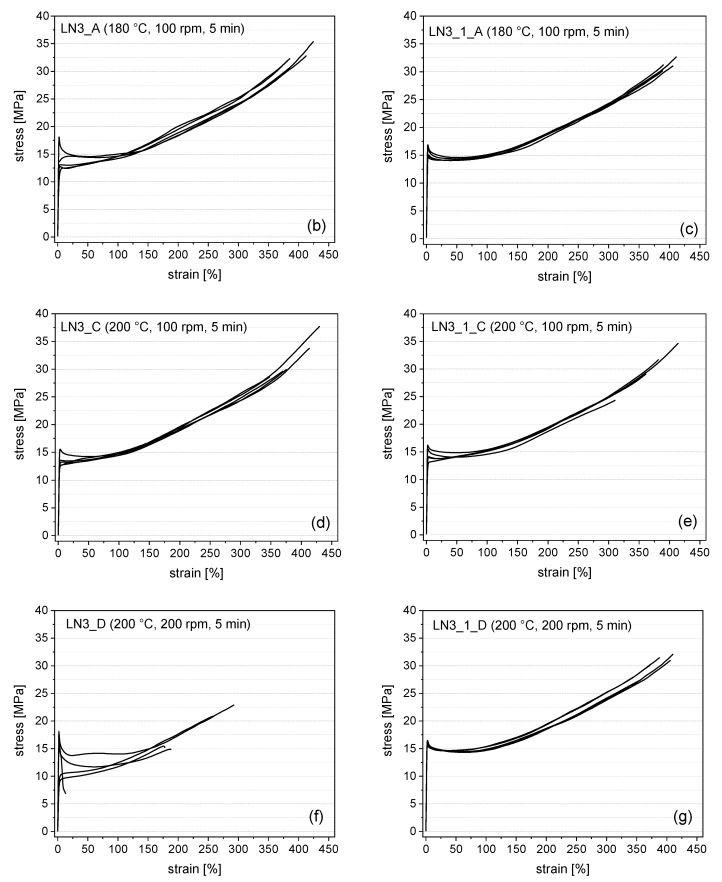
Stress-strain diagrams of pure LN3 and respective composites with 1 wt% of MWCNTs: (**a**) LN3_neat, (**b**) LN3_A, (**c**) LN3_1_A, (**d**) LN3_C, (**e**) LN3_1_C, (**f**) LN3_D, and (**g**) LN3_1_D.

To study the influence of the carbon nanofiller on the mechanical properties of the triblock copolymer, tensile tests were performed on specimens cut from compression-molded plates, which were processed using granules as supplied by the manufacturer. The mechanical characteristics of the so-called LN3_neat sample, compared to the LN3 types and their respective LN3/MWCNT composites containing 1 wt% MWCNTs processed under conditions A, C, and D, are presented in Figure 11. To provide further information about the deformation behavior of each sample, the stress-strain diagrams of all samples are available in the Appendix A in Figure A1. Each diagram contains the curves of five tested specimens. LN3_neat exhibits very ductile behavior, with large strain at break of ~ 415 %, but also large average value of Young’s modulus of ~915 MPa (Figure A1a), indicating high resistance against elastic deformation, i.e., high stiffness. Depending on the orientation of the polymer chains and hence on the alignment of the lamellae within the tested specimens of sample LN3_neat, the Young’s modulus and yield point exhibited significant variations. This is also evident from the large standard deviations of these mechanical values, as shown in Figure 11a,b. As discussed by Allan et al. [53], samples with orientation of the lamellae parallel to the deformation direction exhibit the highest Young’s modulus, followed by samples with perpendicular direction of the lamellae. Lowest moduli occur if the lamellas are aligned at an angle of 45 ° to the deformation direction. Structural orientations of these samples were mainly caused by the extrusion process within the granules of the extruded strands, followed by the subsequent compression molding process. Thus, depending on the orientation of the lamellae within the deformed region of the specimens, the nature of the material ranges from thermoplastic with distinct yield point and high ductility to thermoplastic elastomeric behavior indicated by the absence of a yield point and large strain prior to failure. Detailed research on the influence of ram extrusion with different shearing rates on structure and mechanical properties of such triblock copolymer was published by Mahmood et al. [54] but is not the focus of this study.

Similar mechanical behavior like that of LN3_neat could be observed for the melt-extruded samples of LN3_A and LN3_C (Figure A1b,d). These samples exhibited large strain at break and tensile strength values similar to the neat LN3 (Figure 11c,d), but lower stiffness, due to larger influence of processing-induced orientations of the lamellar morphology. However, in sample LN3_D the ductility has been observed to reduce significantly, and large variations in the magnitudes of the mechanical properties implied defect areas such as those originating from voids and melt inhomogeneities in the pressed plates.

Interestingly, the mechanical property values of the corresponding LN3/MWCNT composites with 1 wt% nanofiller content are stabilized compared to the pure LN3 samples. By adding MWCNTs, structural orientation effects decreased and there were no more large fluctuations of the Young’s modulus within a sample series (Figure 11a). The composites showed a stable yield point (Figure 11b) accompanied with the unaltered tensile strength (Figure 11c) and high elongation at break of the pure BCPs (Figure 11d).

Furthermore, a slight influence of processing conditions on the mechanical properties could be observed from the data shown in Figure 11. With increased screw speed (processing condition B and D), an enhancement in Young’s modulus and yield stress of the composites LN3_1_B and LN3_1_D compared to LN3_1_A and LN3_1_C, respectively, could be observed. This can be attributed to the better dispersion and more homogeneous distribution of the CNTs due to the higher shear forces applied during melt mixing, which was indicated by the lower values of the agglomerate area ratio and their variations (Figure 10). Similar observations were made by Arrigo et al. [55] in composites of SBS and modified MWCNTS containing COOH groups, which were melt mixed in a Brabender mixer at 50 rpm and 100 rpm. The study also reported a significant influence of processing temperature on the mechanical properties, an observation that could not be validated from the results obtained in the context of the present work.

## 4. Conclusions

Investigations of the structure, mechanical, and electrical properties of linear triblock copolymer/MWCNT composites revealed very complex interrelations between processing conditions and structural development. Pretreatment of the used granules, the melt extrusion process in the micro compounder, and the compression molding technique have a significant impact on the orientation of the lamellas, which in turn clearly controls the mechanical properties. Via the addition of MWCNTs, the alignment effects of the lamellar structure decrease significantly as evaluated by SAXS. The MWCNTs could be well dispersed within the BCP matrix and tend to be anchored in the PB-rich phase. However, selective localization of the nanotubes in one of the BCP phases could not be clearly identified. It is apparent that MWCNTs, despite their shortening, are still too large to be fully embedded in the lamellas. However, by the addition of only 1 wt% MWCNTs the triblock copolymers are found to be electrically conductive. The excellent mechanical property profile of the BCP is retained, making these nanocomposites very attractive for the development of new functional materials. In order to increase the possibility of selective localization of the CNTs in one of the BCP phases, more favourable size ratios between nanotubes and block copolymer phases must be created by, for example, expanding one of the BCP phases by adding a corresponding homopolymer [35,56] or using even shorter CNTs. Furthermore, the compatibility between the components can be increased by appropriate functionalization of the CNTs. These approaches are the subject of our further research and will be discussed in future papers.

## Figures and Tables

**Table 1:** Molecular characterization of the block copolymer used in this study (LN3), Data from [5].

Block Composition	*M_n_* (g/mol)	*M_w_/M_n_*	Total PS content (wt%)	PS_1_/PS-co-PB/PS_2_ (wt%)	S/B Ratio in the Random Copolymer Block
PS_1_/PS-co-PB/PS_2_^1^	110 000	1.21	75	12/49/39	1:1

^1^ Scheme of Block architecture:

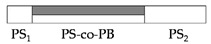

**Table 2:** Processing conditions of LN3 and LN3/multiwalled carbon nanotubes (MWCNT) composites.

Sample.	MWCNT Content (wt%)	Melt Mixing Parameter	
		Temperature (°C)	Screw Speed (rpm)
LN3_A	-	180	100
LN3_C	-	200	100
LN3_D	-	200	200
LN3_1_A	1	180	100
LN3_1_B	1	180	200
LN3_1_C	1	200	100
LN3_1_D	1	200	200
LN3_0.1_D	0.1	200	200
LN3_0.3_D	0.3	200	200
LN3_0.5_D	0.5	200	200

**Table 3:** Long period and domain sizes of the lamellar morphologies of LN3 and LN3/MWCNT composites evaluated from TEM images.

Sample	MWCNT Content (wt%)	Long Period (nm)	Domain Size (nm)	
			PS-rich Domain	PB-rich Domain
LN3_A	-	22.0 ± 5.7	12.8 ± 3.1	10.3 ± 2.8
LN3_C	-	32.0 ± 4.2	17.7 ± 3.4	15.1 ± 2.4
LN3_D	-	27.5 ± 7.3	17.7 ± 5.1	10.2 ± 3.1
LN3_1_A	1	22.4 ± 4.4	11.1 ± 3.2	11.2 ± 3.8
LN3_1_B	1	28.7 ± 4.5	14.1 ± 2.7	13.9 ± 3.5
LN3_1_C	1	24.2 ± 4.4	12.4 ± 2.3	11.2 ± 3.1
LN3_1_D	1	21.1 ± 6.0	10.6 ± 2.7	9.4 ± 3.1
LN3_0.1_D	0.1	15.7 ± 2.3	9.1 ± 1.8	7.4 ± 2.0
LN3_0.3_D	0.3	25.6 ± 4.0	10.8 ± 2.4	14.8 ± 2.9
LN3_0.5_D	0.5	18.0 ± 2.1	8.4 ± 1.8	9.9 ± 1.6
